# The Role of Mitochondria in Metabolic Syndrome–Associated Cardiomyopathy

**DOI:** 10.1155/2022/9196232

**Published:** 2022-06-23

**Authors:** Jiayu Li, Jingye Li, Yijun Chen, Wenyu Hu, Xuhe Gong, Hui Qiu, Hui Chen, Yanguo Xin, Hongwei Li

**Affiliations:** ^1^Department of Cardiology, Cardiovascular Center, Beijing Friendship Hospital, Capital Medical University Beijing, China; ^2^Department of Gastroenterology, Beijing Friendship Hospital, Capital Medical University, Beijing, China; ^3^Department of Cardiology, The First Affiliated Hospital of China Medical University, Shenyang, Liaoning, China; ^4^Beijing Key Laboratory of Metabolic Disorder Related Cardiovascular Disease, Beijing, China; ^5^Department of Geriatrics, Cardiovascular Center, Beijing Friendship Hospital, Capital Medical University, Beijing, China

## Abstract

With the rapid development of society, the incidence of metabolic syndrome (MS) is increasing rapidly. Evidence indicated that patients diagnosed with MS usually suffered from cardiomyopathy, called metabolic syndrome–associated cardiomyopathy (MSC). The clinical characteristics of MSC included cardiac hypertrophy and diastolic dysfunction, followed by heart failure. Despite many studies on this topic, the detailed mechanisms are not clear yet. As the center of cellular metabolism, mitochondria are crucial for maintaining heart function, while mitochondria dysfunction plays a vital role through mechanisms such as mitochondrial energy deprivation, calcium disorder, and ROS (reactive oxygen species) imbalance during the development of MSC. Accordingly, in this review, we will summarize the characteristics of MSC and especially focus on the mechanisms related to mitochondria. In addition, we will update new therapeutic strategies in this field.

## 1. Introduction

Along with the changes in lifestyle and population aging, the incidence of a series of metabolic disorders, such as obesity, diabetes mellitus (DM), insulin resistance, dyslipidemia, and hypertension, is increasing worldwide [1, 2]. People suffering from these disorders are diagnosed with metabolic syndrome [3]. Current evidence indicated that these patients are twice as at risk to develop cardiovascular diseases (CVD) as those without. Metabolic syndrome–associated cardiac diseases (MSCDs) include cardiomyopathy, coronary artery disease (CAD), left ventricular hypertrophy (LVH), and systolic and/or diastolic contractile dysfunction with an increased incidence of heart failure (HF) [4]. However, the underlying mechanisms of this pathological process are not clear yet.

Mitochondria is the most important organelle involved in cellular or tissue metabolic physiological processes [5]. Since mitochondrial homeostasis is vital to maintaining physical cellular activity, mitochondrial quality control disorders through various processes such as proteostasis, mitochondrial dynamics, and mitophagy could lead to the development of metabolic syndrome–related cardiomyopathy (MSC) [6]. In this review, we would first introduce the changes in MSC, and then we will review the role of dysregulated mitochondrial homeostasis in cardiomyopathy induced by metabolic syndrome. Finally, as healthy mitochondria may be essential for normal cardiovascular function, we will focus on the underlying therapeutic strategies targeting mitochondria in the development of MSC.

## 2. Definition of Metabolic Syndrome–Related Cardiomyopathy

Patients suffering from obesity, insulin resistance, dyslipidemia, and diabetes are at high risk to develop cardiomyopathy [7, 8]. If patients with diabetes or obesity suffered cardiomyopathy, independently of hypertension, or CAD, it can be termed as metabolic syndrome-associated cardiomyopathy. We will discuss obesity-related and diabetes-related cardiomyopathy as the most-studied situations in detail in this section. Diabetic cardiomyopathy (DCM) is first described in the 1970s in some DM patients with no attributable factors [9]. The following Framingham study identified DM as an independent risk factor for cardiomyopathy regardless of comorbidities such as hypertension, CAD, and dyslipidemia [10]. Lately, clinical physicians found that many patients develop DCM with well-controlled DM [11, 12]. It's reported that about 60% diabetic patients suffered unexplained myocardial hypertrophy and fibrosis, which is characterized by left ventricular diastolic dysfunction. This situation emphasized the urgency to elucidate the underlying molecular mechanisms driving this pathological condition [13, 14, 15]. Obesity, insulin resistance, and dyslipidemia could also initiate similar cardiomyopathy even in the absence of DM, which is defined as obesity-related cardiomyopathy [16, 17, 18]. Although there may be distinct underlying mechanisms of cardiac structural and functional abnormality between obesity and DM, they shared similar dysregulated metabolism in the development of cardiomyopathies [16].

## 3. Heart Changes in MSC Structure, Function, and Metabolism

### 3.1. Structural Change

**Figure 1:**
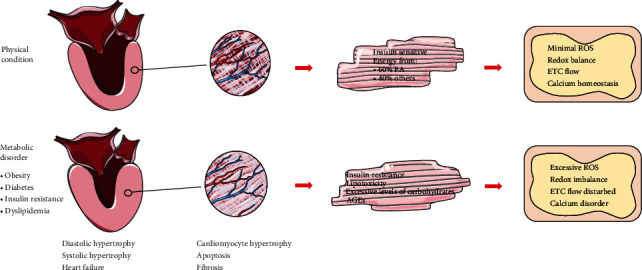
The structural changes of MSC and the mitochondrial changes. Under physical condition, the cardiomyocytes generate energy about 40% via FA metabolism, and mitochondrial hemostasis is regulated via various mechanisms. Obesity, insulin resistance, diabetes, and dyslipidemia cause cardiomyopathy, characterized by thickness and stiffness of the ventricular wall (interstitial fibrosis and cardiomyocyte hypertrophy), cell death, and eventually contractile dysfunction. The accumulation of toxic metabolism intermediates and leads to decreased oxygen utilization induced by mitochondrial dysfunction (reactive oxygen species (ROS) generation, altered calcium, and ETC distribution).

The Framingham study found that diabetes may be an independent factor of LVH and cardiac stiffness. Lately, another study found that increased left ventricular mass was only identified in diabetic patients but not in those with impaired fasting glucose concentrations [19], indicating that cardiac dysfunction did not present in the early stage but was a consequence of chronic hyperglycemia. In the following research, Eguchi and colleagues also uncovered the close interrelation between DM and propensity of LVH [20]. Similarly, alterations in the left ventricular structure are also found in obese patients without hypertension and diabetes, in which condition the heart may develop a thicker left ventricular wall, elevated left ventricular mass, left ventricular dilatation, and significant left ventricular remodeling (Figure 1), [21, 22].

Fibrosis is also a key characteristic in MSC [15, 23], characterized by diastolic restriction, other features including decreased early diastolic filling and increased atrial filling, and isovolumetric relaxation time [24, 25]. One of the dominant features of the diabetic rat model is the increased expression levels of TGF-*β*1 and connective tissue growth factor, which could attribute to the deposition of collagen in the heart [26, 27]. According to Shimizu et al., diabetes-associated myocardial fibrosis comes with increased deposition of both types I and III collagen. In DM patients presenting diastolic dysfunction, the deposit of procollagen type I carboxyl-terminal peptide levels increased significantly [28]. Myocardial tissue from diabetic patients showed dominant interstitial fibrosis and perivascular fibrosis [9]. In diabetic hearts, the increased matrix metalloproteinase (MMPs) also causes cardiac extracellular matrix disorder and increases connective tissue content [27]. Left ventricular remodeling, including increased wall thickness and ventricular dilatation, is a common result of hemodynamic changes induced by obesity [29, 30]. Both eccentric and concentric LV hypertrophy were found in obese individuals. In addition, the right ventricle is also affected adversely by obesity; the manifestation of right ventricular hypertrophy and final dilatation were reported in heart failure induced by obesity [31, 32]. Histologic findings revealed that increased heart weight and left and right ventricular hypertrophy are proportional to the development of obesity [33].

### 3.2. Functional Change

Diastolic dysfunction is a significant clinical characteristic of MSC. Schannwell et al. [34] indicated that patients with diabetes demonstrated cardiac diastolic dysfunction without known coronary artery diseases [35]. According to previous evidence, the incidence of diastolic dysfunction varies from 30% to 75% in patients with well-controlled DM but without coronary artery diseases [36].

In addition to diabetes, there is also evidence finding that LV diastolic dysfunction is associated with obesity. Berkalp et al. [37] reported diastolic dysfunction diagnosed by load-dependent spectral Doppler in young obese women. Besides, body mass index (BMI) was an independent predictor of diastolic dysfunction according to a specific Doppler-derived load-independent measurement [38, 39, 40]. Various studies demonstrated that patients with MSCs were usually characterized by increased stiffness of ventricular myocardium and developed systolic dysfunction followed by diastolic dysfunction [41, 42, 43].

### 3.3. Metabolic Change

The heart is a high-energy-consuming organ. In physical conditions, FA (FA) contributes 60% of energy for the adult heart, with the rest 40% supported by the oxidation of other fuel substrates (Figure 1**)**, [44]. The cardiac mitochondria are the energy house. Thus, the heart muscle is vulnerable to mitochondria damage. Sarah et al. first reported the increased mitochondrial fission in the myocardium of diabetic patients [45]. Lately, other studies found that FA metabolism in the hearts of asymptomatic diabetic patients significantly increased to compensate for the decreased respiratory capacity, suggesting that changes in mitochondrial metabolism are detrimental to the heart [46, 47].

### 3.4. Lipotoxicity

**Figure 2:**
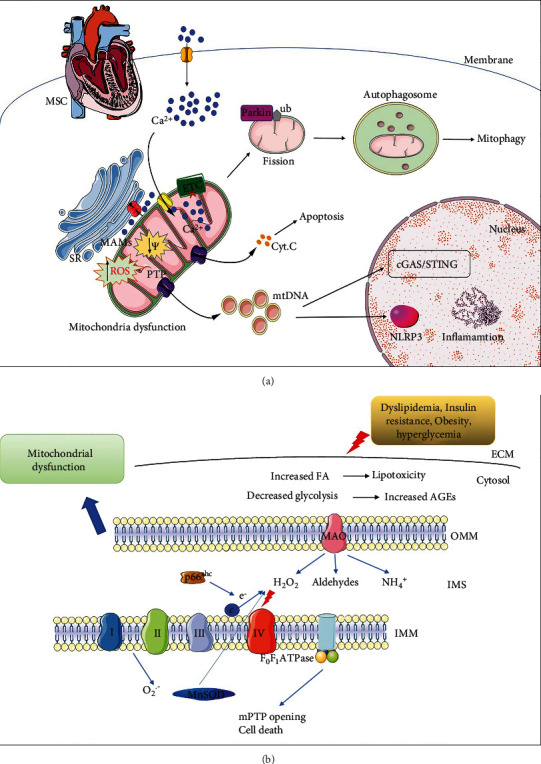
Mitochondrial signaling in metabolic syndrome-related cardiomyopathy. (a) Dyslipidemia, insulin resistance, obesity, and hyperglycemia could cause cardiomyocytes altered calcium homeostasis, including the cytoplasmic calcium, SR, and mitochondrial calcium. Damaged mitochondria undergo fission and are finally cleaned by mitophagy. In addition, the damaged mitochondria exert mPTP opening and cytochrome c release, inducing apoptosis. (b) Dyslipidemia, insulin resistance, obesity, and hyperglycemia lead to lipotoxicity and accumulation of AGEs, leading to the activation of mitochondrial ROS-producing enzymes. p66Shc is activated and catalyzes the formation of hydrogen peroxide (H_2_O_2_). Increased ROS could disturb the mitochondrial oxidation respiratory chain, followed by cell death.

Deleterious effects of lipid accumulation in non-adipose tissues are usually termed lipotoxicity [47], which may be caused by the accumulation of products from incomplete oxidation, such as acylcarnitines and ROS (Figure 2(a))[22]. Kim and colleagues [48] found that the level of even-chain acylcarnitines increased in skeletal muscle, correlated with increased mitochondria-mediated incomplete oxidation. In the following analysis, Palomer et al. found that PPAR, a mitochondrial biogenic receptor, was involved in lipid metabolism [49], and increased PPAR expression reduced the level of incomplete oxidation of fatty acids in myoblasts. PPAR is also vital to regulating the transcription of FA metabolism via stimulating related transcriptional genes, such as CD36 [50] and pyruvate dehydrogenase kinase 4 [51]. In addition, glycogen synthase kinase 3*α* (GSK-3*α*) is a sensor, inducing lipid uptake and accumulation via PPAR activation [52]. Patients with diabetic cardiomyopathy received agents targeted at GSK-3*α* or PPAR could demonstrate alleviated cardiac lipotoxicity. Ceramides belong to the lipid molecule families, including sphingosine and fatty acid [53]. PPAR*α*-related agents such as K-877, a novel selective PPAR*α* modulate, have a great triglyceride-lowering activity [54]. K-877 could upregulate fibroblast growth factor 21 and lipoprotein lipase levels, which have been identified in various clinical trials [55, 56, 57, 58].

Ceramides could activate mitochondria-related apoptotic pathways via increased mitochondrial membrane permeability, leading to increased ROS levels through mitochondrial electron transport distributions, followed by apoptosis and insulin resistance [16]. As a saturated long-chain fatty acid, palmitate could induce apoptosis of rat neonatal cardiomyocytes via the decrease of mitochondrial cardiolipin [59]. Calcium and ROS disorders could initiate long-chain FA accumulation and alter mitochondrial membranes by changing phospholipid composition, followed by the release of cytochrome c from the mitochondria and final apoptosis [60, 61]. To protect the heart from lipotoxicity damage, mitochondrial uncoupling and futile cycling may work as major adaptive mechanisms [22, 62]. Uncoupling proteins (UCPs) work as FA anion exporters, exporting fatty acids out of the mitochondrial matrix by a flip-flop mechanism, reducing the proton gradient, followed by a subsequent decrease in oxidative stress [63]. Similarly, mitochondrial thioesterase 1 could hydrolyze fatty acyl-CoA in the mitochondrial matrix and release FA anion and CoASH, which is essential for the increased oxidation states [64, 65, 66].

### 3.5. Mitochondria-Mediated Redox Status in DCM

In mitochondrial electron transfer chain (ETC), electrons transfer from reduced to oxidized submits, finally forming oxygen, which is a classic example of redox reactions [11]. NAD^+^/NADH and NADP^+^/NADPH, TrxSS/TrxSH2, and GSSG/GSH are important redox couples to maintain mitochondria function [67]. The NADPH/NADP^+^ redox couple is an important antioxidant defense mechanism by transferring electrons to glutathione and thioredoxin systems, both of which are scavengers of hydrogen peroxide (H_2_O_2_) [68]. The mitochondrial redox states of the NADH/NAD^+^ and NADPH/NADP^+^ redox couples have different metabolic roles [69]. ETC (via complex I) and the antioxidant system received electrons from fuel substrates with support from NADH/NAD^+^ [11]. The NADPH/NADP^+^ couple offers electrons to GSH reductase and thioredoxin reductase 2, maintaining their reduced states [11]. NAD^+^ participates in cardiac mitochondrial fitness by regulating mitochondrial biogenesis and dynamics. In addition, NAD^+^ is also a substrate of various enzymes, such as the family of sirtuins (SIRTs) [70].

The interaction between NAD^+^/NADH redox and mitochondria bioenergetics is critical to maintaining normal cardiac function as an essential regulator in the heart [71]. The early stage of obesity- or diabetes-induced cardiomyopathy is usually accompanied by highly reduced NAD^+^/NADH redox couple. Furthermore, the well-controlled glucose levels in diabetic patients usually have lower cardiac mortality, indicating that hyperglycemia is a detrimental factor. Previous evidence proved that some non-energy pathways, such as the formation of the late glycation end products, activation of the hexosamine pathway, and activation of the polyol pathway, are central factors contributing to diabetic chronic microvascular complications, mediating cellular redox changes [72, 73]. Aldose reductase converted glucose to sorbitol via NAD^+^/NADH redox, which has been extensively studied in microvascular complications [74]; however, its impact on diabetic myocardium was not fully elucidated. In high glucose conditions, increased diacylglycerol caused protein kinase C activation and final cardiomyopathy [75]. Glucose-derived methylglyoxal could target mitochondrial proteins and components of calcium cycling, causing metabolic disorders in the heart [76]. O-glcNAcylation is an important post-translational modification via O-GlcNAc transferase and substrate from the hexosamine pathway [77], causing contractile dysfunction, epigenetic modifications, and genetic changes in the diabetic heart.

Compared with glucose, excessive FA damaged mitochondrial energy via increasing NADH, which promoted ETC electron flow, mitochondrial membrane hyperpolarization, and superoxide formation [70]. Excessive circulating FA seems to be the trigger of the change of mitochondrial NAD^+^/NADH redox state during energetic challenges [11]. Decreased circulating FA could activate NAD^+^-dependent mitochondrial SIRTs to deacetylate ETC proteins and reverse diastolic dysfunction in DCM [78]. Besides the regulation of the energetic metabolism of NAD^+^/NADH redox couple, various researches focused on the role of increased acetyl-CoA derived from the oxidation of excessive fuels [79]. In diabetic conditions, increased mitochondrial FA oxidation is usually accompanied by a high acetylation level of related enzymes regardless of unchanged SIRT3 protein abundance [80]. Similarly, a high-fat diet (HFD) led to increased FA oxidation enzyme acetylation and long-chain acyl-CoA dehydrogenase (LCAD) activation, enhancing cardiac FA oxidation [81]; thus, the increased NAD^+^/NADH redox state and decreased FA oxidation enzyme acetylation could improve heart function in diabetes [82]. SIRT3 is downregulated followed by decreased NAD^+^/NADH ratio in HFD, the acetylation levels of heat shock protein 10 increased, and induced mitochondria FA oxidation [83]. In the diabetic heart, chronic hyperglycemia could create an excessive oxidative redox environment and disturb mitochondrial antioxidant scavengers via non-mitochondrial mechanisms [84]. In physical conditions, the glutathione and thioredoxin redox couples are the major scavengers of H_2_O_2_. However, during hyperglycemia, this scavenger system becomes more oxidized, and short-term mitochondrial palmitate oxidation could provide reduced NADH, normalizing the redox status and providing a more energetic budget to improve the function of the diabetic heart [85]. Chronic exposure to hyperglycemia and excessive FA exhibit multiple metabolic effects. Excessive FA oxidation could generate a high level of acetyl-CoA and NADH, which could activate PDK4, inhibiting pyruvate oxidation [86]. Nicotinamide mononucleotide (NMN) is a kind of intermediate in NAD+ biosynthesis, which was reported to improve multi-organ insulin sensitivity [87]. NMN can improve various metabolic complications related with obesity, although this effect on many other obesity-related metabolomic complications is yet to be explored [88]. Keio University and Washington University School of Medicine of St. Louis carried out a collaborative preclinical trial of NMN to observe the efficacy of NMN on aging [89], hoping that there will be evidence in the field of cardiovascular diseases in the future.

## 4. Mitochondrial Signaling in Metabolic Syndrome-Related Cardiomyopathy

### 4.1. Mitochondrial Biogenesis

The biogenesis of mitochondria is associated with rapid cell growth and proliferating cells [90]. It is reported that peroxisome proliferator-activated receptor gamma coactivator-1*α* (PGC-1*α*) is an important transcriptional activator for the regulation of mitochondrial function [91, 92], via the activation of various transcription factors that mediated the gene expression both in the nucleus and mitochondria [93]. PGC-1*α*-driven mitochondrial biogenesis is indispensable for normal cardiac and skeletal muscle contraction and relaxation by regulating mitochondrial biogenesis, such as regulating fatty acid metabolism and oxidative phosphorylation, increasing mitochondrial number, and enhancing mitochondrial respiration ability [94]. EET analog is an activator of PGC-1*α* pathway; various evidence indicated that increased levels of EETs are beneficial in the prevention of cardiac consequences besides diabetic treatment [95, 96].

Changes in mitochondrial biogenesis have been reported in patients diagnosed with metabolic syndrome and diabetes [97, 98]. Studies have shown that diabetic cardiomyopathy is characterized by the accumulation of toxic materials, such as long-chain acyl-CoA and acylinosine, which influence the mitochondrial ATP/ADP ratio and reduce mitochondrial metabolic rates [99]. A reduction in mitochondrial biogenesis is observed in metabolic syndrome models, which is consistent with low ATP levels and faulty electron transport. Increased cardiac energy demand modulates gene expression in nuclear and mitochondrial DNA and maximizes the ability of mitochondria to perform oxidative phosphorylation. In the setting of obesity and insulin resistance, nutrient excess, coupled with reduced energy requirements, leads to reduced mitochondrial DNA gene expression, which in turn leads to reduced oxidative phosphorylation [100].

### 4.2. Mitophagy

It is necessary to remove dysfunctional mitochondria from cardiomyocytes to maintain myocyte survival. Mitophagy is an efficient way to remove damaged mitochondria. Many pathways have been reported to regulate mitophagy (Figure 2(a)). The PINK/Parkin pathway is the first pathway reported to clean damaged mitochondria. As a serine/threonine kinase, PINK1 is imported to healthy mitochondria and degraded by related proteases [101]. With the decrease of mitochondrial membrane potential, PINK1 could accumulate on the outer membrane of mitochondria, followed by the phosphorylation of the membrane proteins. Mitofusin 2 is a mitochondrial dynamic protein, which could recruit Parkin to the mitochondria [102]. Parkin is an E3 ubiquitin ligase; upon translocating to mitochondria, Parkin could ubiquitinate mitochondrial proteins, promoting the recruitment of autophagosome and initiating mitophagy [103]. Studies including obese or diabetic patients found that the transcript levels of PINK1 are decreased in skeletal muscle, suggesting that mitophagy regulated by the PINK1/Parkin pathway is inhibited [104]. In addition, Parkin dysfunction may lead to the loss of pancreatic *β* cells and the development of insulin deficiency [105]. Bharath and colleagues found that in diabetic mice, the level of p53 increased in *β*-cells, inhibiting Parkin-mediated mitophagy [106]. However, in p53 deficient mice, restored mitophagy protected *β*-cell loss induced by streptozotocin (STZ). While the PINK1/Parkin pathway plays an important role in mitochondrial homeostasis in the heart, its role in diabetic cardiomyopathy remains unclear.

The role of mitophagy and its underlying mechanisms in the diabetic heart remain largely unknown [107]. PINK1/Parkin pathway is inhibited in the hearts of various diabetic animals, indicating that mitophagy is closely related to diabetic hearts [108]. Beclin 1 or Atg 16L1 deficient mice could restore the PINK1/Parkin pathway, alleviating diabetic heart function [109]. Autophagy and mitophagy performed differently in the pathologic process of diabetic hearts. In the early stage of the diabetic heart, autophagy is inhibited, but mitophagy decreased in the advanced stage [110]. Thus, it is important to separate mitophagy from autophagy to uncover the underlying mechanisms of diabetic hearts. Xu et al. found that the expression of Rab 9 increased in diabetes, mediating damaged mitochondria degradation [111]. This result indicated that alternative autophagy may be increased to initiate mitophagy, compensating canonical autophagy and finally protecting the diabetic heart. However, other autophagic pathways, such as Beclin-independent and Atg7-dependent pathways, are necessary to be explored.

### 4.3. Mitochondrial-Derived ROS

The generation and clearance of ROS are important in various pathological conditions [112]. Current evidence found that ROS could damage numerous molecules including proteins, amino acids, lipids, and DNA (Figure 2(a)). Hyperglycemia produced mitochondrial ROS in various ways [113], such as GAPDH inhibition, activation of the polyol pathway, formation of AGEs, and glucose auto-oxidation; in turn, these pathways could exacerbate ROS generation. For example, in the polyol pathway, NAPDH is indispensable for GSH generation, and the increased AGEs lead to ROS formation [114]. Inhibition of the formation of AGEs or blocking its receptors significantly improved the cardiac function of DCM. In addition, Kaludercic and colleagues found that in diabetic mice, increased expression of p53 led to disturbed mitochondrial respiration and increased oxidative stress, followed by cytochrome c release from mitochondria [113]. In addition to hyperglycemia, lipotoxicity could also lead to oxidative stress via the oxidation of free fatty acids [115]. Mitochondrial ROS could activate the NLRP3 inflammasome, promoting cardiac fibrosis [116]. Moreover, hyperglycemia, as well as inflammation, exhibits synergistic effects, enhancing ROS generation and final cell death [113].

In the diabetic heart, ROS is involved in various processes, such as inflammation, angiogenesis, and apoptosis to mediate fibrosis and hypertrophy [117]. In diastolic cardiac dysfunction, ROS could target sarcomere proteins, leading to the accumulation of oxidative thick and thin filaments, demonstrating LV stiffness [118, 119, 120, 121]. Evidence indicated that oxidation and disulfide bridge formation led to increased cardiac stiffness [122]. Recently, Grutzner et al. found that ATP synthase was cleaved by calpain-1 induced by hyperglycemia, resulting in increased ROS generation and deteriorated DCM [123]. Moreover, mitochondrial ROS could increase the propensity of mitochondrial permeability transition pore (mPTP) opening and apoptosis [124].

P66Shc is another important source of ROS in mitochondria (Figure 2(b)) [125, 126]. Epigenetic modification of the p66Shc promoter is important for its expression [127]. Hyperglycemia could induce p66Shc phosphorylation and translocate to mitochondrial intermembrane space, resulting in the formation of H_2_O_2_ [128, 129]. Meanwhile, p66Shc could also amplify oxidative stress by activating mitochondrial NOX or by downregulating the synthesis of other antioxidant enzymes [130]. Accordingly, cells and mice lacking p66Shc show reductions in markers of oxidative stress [131]. Phosphorylation by PKC is required for p66Sshc translocating to mitochondria in hyperglycemia conditions [132], indicating that p66Shc may play a role in DCM [132]. Diabetic p66Shc^−/−^ hearts displayed preserved cardiac progenitor cell replication and LV end-diastolic pressure [133]. Szeto-Schiller peptide SS31 is a free-radical scavenger that protects from cardiac hypertrophy, via preventing cardiolipin from converting cytochrome c into a peroxidase or protecting mitochondrial cristae structure [134, 135].

Mitochondria dynamics are also reported to be associated with ROS formation that may reciprocally modulate each other. In diabetic mice and diabetic patients, cardiomyocytes demonstrated increased ROS, mitochondrial fragmentation, cristae disruption, and swelling [136]. Dillmann et al. found that c ronic-hyperglycemia–induced mitochondrial fragmentation could be reversed by antioxidants, indicating that ROS are causally related to mitochondrial morphology. This partially explained the underlying mechanism of antioxidants treatment in DCM [137]. Mitochondrial ROS reduction via cardiac-specific Mn-SOD overexpression or stimulation of the AMPK pathway will rescue mitochondria from fatty acid- or hyperglycemia-induced events [138]. Brownlee's group reported that high intracellular glucose disturbed mitochondrial respiration, leading to mitochondrial membrane hyperpolarization, and ETC superoxide production [139, 140]. Interventions such as ETC complex II inhibition could decrease the levels of mitochondrial ROS and prevent the activation of the hexosamine pathway, AGEs formation, and sorbitol accumulation [112]. Additionally, Brownlee et al. also found that hyperglycemia could not lead to increased ETC superoxide production in Rho endothelial cells lacking ETC function [141].

High glucose induced cardiomyocyte damage via a similar mechanism [142, 143, 144]. Current evidence proved that mitochondrial ROS could be generated from cardiac lipotoxicity [145]. Palmitate stimulated ROS generation, leading to mitochondrial fission via Drp1 phosphorylation and OPA1 degradation [145]. This process could be amplified by increased ROS levels and vice versa [146]. In addition, mitochondrial oxidative stress promoted the occurrence of post-translational modifications, such as methylglyoxal formation and O-GlcNAcylation, contributing to the impairment of heart function [147, 148]. O-GlcNAc transferase enzyme located in the IMM could interact with complex IV of the respiratory chain in physical conditions. Hyperglycemia could alter the function of the respiratory chain in mitochondria via dysregulation of O-GlcNAcylation [147].

In the diabetic rat model, the interaction between O-GlcNAc transferase and complex IV is impaired, leading to the disorder of complex IV activity and reduction of mitochondrial membrane potential. Many mitochondrial proteins are O-GlcNAcylated, such as Drp1 and OPA1, resulting in mitochondrial dysfunction [149]. On the other hand, methylglyoxal modifications also cause calcium disturbance, followed by mitochondrial damage [150], calcium-related proteins including ryanodine receptor 2 and SERCA2a that may transform to methylglyoxal forms and cause mitochondrial dysfunction [76]. Collectively, these studies emphasized the critical role of ETC-derived superoxide in diabetic conditions. TACT (trial to assess chelation therapy) is a clinical trial which uses edetate disodium to inhibit conversion of H_2_O_2_ to peroxynitrite. It came out that the chelators could significantly reduce cardiac events especially in diabetic patients [151].

### 4.4. Monoamine Oxidases

Monoamine oxidases (MAOs) are flavoenzymes localized at the outer mitochondrial membrane [152]. There are two isoforms of MAOs (MAO-A and MAO-B), with different characteristics of structures, substrate inclination, and tissue distribution (Figure 2(b)) [153]. Over the last decade, many studies demonstrated that the redox unbalance induced by MAOs promoted the development of cardiovascular diseases [154, 155, 156, 157]. MAOs have been reported to be involved in diabetic complications; Emory et al. found that MAO inhibitors could reverse hyperglycemia conditions and improve cardiac function [158]. Additionally, pioglitazone could function as a specific and reversible MAO-B inhibitor in diabetic patients [159]. In diabetic animal models including mice and rats, MAO inhibition was also proved to reverse DCM function [160, 161]. MAO inhibitors can prevent oxidative changes, followed by diastolic dysfunction, and myocardial fibrosis.

Besides, MAO inhibitors could prevent mast cell degranulation, alleviating cardiac fibrosis in diabetic hearts. Similar pathological processes were also found in cardiomyocytes and mesenchymal stromal cells [160, 161]. According to current studies, the underlying mechanism of MAO toxicity was due to the high level of H_2_O_2_ and aldehyde formation, leading to impaired mitochondrial function. Increased MAO activity induced inflammasome activation and promoted the inflammatory process, while MAO inhibition could also cause mitochondrial dysfunction and ER stress, contributing to DCM [160]. Mitochondria and ER communicate a lot in physical and functional aspects; there is a special domain named mitochondrial associated membranes (MAMs) function as a platform to regulate calcium transport, lipid metabolism, and autophagy flow (Figure 2(a)). MAO is localized at the outer mitochondrial membrane and could interact with proteins in MAMs to regulate ER function [113]. Mitochondrial aldehyde dehydrogenase 2 (ALDH2) is important to mediate mitochondrial metabolism; MAO-related oxidative stress inhibits ALDH2 activity, resulting in the accumulation of toxic aldehydes [162], while increasing ALDH2 activity protects cardiac damage induced by streptozotocin. Some evidence supports that there is a ROS amplification mechanism or so-called the ROS-induced ROS release mechanism, where initial stress triggers ROS production that, in turn, leads to more ROS formation, setting up a positive feedback loop for the ROS-induced ROS release [163, 164]. An example of this is that high glucose in adult cardiomyocytes increased MAO-dependent ROS formation, leading to mPTP opening and ER stress, which may take other processes into this amplification loop [165, 166]. MAO inhibitors, such as iproniazid and nialamide, have been evaluated for their potential effects in patients with heart diseases [167]. In the future, hoping there would be some clinical trials focusing on the applications of MAO inhibitors in metabolic cardiomyopathy.

### 4.5. Calcium Homeostasis

Calcium is required for maintaining the proper function of the heart; abnormal mitochondrial calcium handling contributes to mitochondrial dysfunction in diabetic cardiomyopathy (Figure 2(a))[168]. Diabetic cardiomyopathy could present various degrees of calcium abnormalities; the decreased activity of sarcolemma Na-Ca exchanger in the diabetic heart can decrease the removal of calcium, leading to the increase of intracellular calcium concentration. MCU is an integral membrane protein mediating mitochondrial calcium across the inner mitochondrial membrane [169]. Previous evidence found that MCU protein levels decreased in the heart of diabetic mice; similar results could be observed in mouse neonatal cardiomyocytes under hyperglycemia. The decrease of MCU expression usually coexisted with impaired mitochondria calcium uptake and release in high glucose conditions. Similar results were observed in mitochondria from diabetic hearts [170]. Diaz-Juarez et al. found that hyperglycemia impaired both mitochondrial calcium uptake and release in living cardiomyocytes detected by calcium sensor Pericam [171]. Furthermore, the fact that cardiomyocytes from diabetic mouse hearts or neonatal cardiomyocytes exposed to high glucose and high fat inducing type 2 diabetes demonstrated a significant decrease in mitochondrial calcium [168, 172]. Mitochondrial Ca^2+^ is an important signaling mechanism to regulate mitochondrial energetic activity. Mitochondrial calcium is an important co-factor to regulate the activity of ATP formation and mitochondrial ETC [173]. It is clear that the heart mainly metabolizes fatty acids under physiological conditions [174, 175]. However, in diabetic hearts, the FA oxidation increased with diminished glucose oxidation and decreased mitochondrial calcium. Decreased calcium further caused the decreased activity of PDC [168]. Calcium/calmodulin dependent protein kinase II (CaMKII) is a multifunctional serine/threonine kinase that mediates physiological responses upon acute *β*adrenergic activation [176]. KN-93 is a CaMKII inhibitor; Sommese et al. reported that KN-93 could prevent spontaneous SR calcium release and the following arrhythmias in diabetic rats.

More evidence also found PDC activity decreased in leptin receptor-deficient mice and increased PDC phosphorylation in cardiomyocytes under hyperglycemia. Impaired glucose oxidation will impair cardiomyocytes' contractile function in the following ways: (1) ATP generated from glycolysis is necessary to promote calcium uptake and maintain SR, sarcolemma, and mitochondria dynamic homeostasis [177, 178, 179]; (2) the “oxygen-wasting” effect caused by fatty acids would induce a higher ratio between cardiac oxygen consumption and heart work, especially following *β*-adrenergic stimulation [180]; and (3) decreased ATP generation from glycolysis would damage the integrity of cellular membranes.

### 4.6. Mitochondria Dynamics

Mitochondrial dynamics (Figure 2(a)) is important to maintain mitochondrial homeostasis. The myocardia cell H9C2 exposed to hyperglycemia demonstrated a significant mitochondrial fragmentation [181], leading to mitochondrial ROS accumulation and final cell apoptosis [182]. Furthermore, these events could be restored by the inhibition of Drp1, indicating that cell apoptosis is mitochondrial dynamics dependent. Moreover, Watanabe et al. reported that Drp1 could interact with ROS, inducing mitochondrial dysfunction and inhibiting the insulin signaling pathway. Antioxidants could partially overcome this process [183]. Also, Makino et al. found mitochondrial fission in endothelial cells from diabetic hearts; further exploration displayed reduced OPA1 and increased Drp1 [184], which could be abolished by antioxidants treatment. This evidence suggested a closed relationship between oxidative stress and mitochondrial dynamics in endothelial cells [185].

Consistently, neonatal cardiomyocytes suffered high glucose presenting mitochondrial fission and decreased mitochondrial membrane potential [186]. In this process, the expression of mitochondrial fusion proteins (OPA1 and Mfn1/2) decreased, and the fission-related proteins (Fis 1 and Drp1) increased significantly. This situation could be restored via overexpression of OPA1 or decreased expression of Drp1[184]. Recently, Parra et al. reported the relationship between mitochondrial dynamics and insulin's impact on cardiomyocyte metabolism [187]. Meanwhile, treating cardiomyocytes with insulin could increase OPA1 levels and promote mitochondrial fusion, increasing the production of ATP [185]. These findings are consistent with those reported by Quirós et al.; they found that OPA1 disturbance causes insulin resistance, impaired glucose homeostasis, and disordered thermogenesis in *Oma1* knockout mice [188]. *Oma1^–/–^* mice developed metabolic disorders after HFD, highlighting the role of OPA1 in regulating mitochondrial function [189]. And Parra et al. also reported that the impact of insulin on mitochondrial metabolism was impaired in OPA1^−/−^ and Mfn2^−/−^ cells [187]. Clinically, in diabetic individuals, impaired mitochondrial functions and diminished levels of OPA1 and Mfn2 were also observed [190, 191, 192]. However, whether mitochondrial dynamic regulation contributes to the cardiac function in diabetic cardiomyopathy is unclear so far. However, current evidence indicates that mitochondrial fragmentation may be a “starting point” of pathological processes involved in cardiac metabolic diseases [193]. Based on this, we hypothesize that ventricular dysfunction occurring in the transition from obesity to DM is caused, at least in part, by the deterioration of cardiac myocyte mitochondrial function. Further studies are necessary to explore the impact of mitochondrial dynamic targets on cardiac performance.

### 4.7. Potential Therapeutic Options

With insight into the mechanisms of MSC, studies were focused on developing novel strategies to protect the heart from obesity and diabetes and to improve the clinical outcomes of these patients. In this section, we will discuss the targets of mitochondria in animal studies and clinical trials.

A series of evidence linked oxidative stress to cardiovascular diseases [194, 195]. Various researches hold the idea that reducing ROS formation could protect the heart from damage caused by diabetes [113]. Nevertheless, many clinical trials failed to identify the protective effects of antioxidants in human subjects. When exploring the certain reason for this situation, it is found that ROS is a double-edged sword; a high level of ROS is deleterious; however, a certain level of ROS is beneficial and required for maintaining physical function. SGLT2 inhibitors are novel antidiabetic agents, exhibiting cardiac protective effects in patients with or without diabetes [196, 197, 198]. Interestingly, human and rodent hearts do not express SGLT2, indicating that the direct cardioprotective effects of SGLT2 inhibitors are independent of their action on SGLT2 [199]. Uthman and colleagues reported that SGLT2 inhibitors could reduce intracellular calcium and sodium levels, improving cardiac mitochondrial function [200]. Empagliflozin could reduce oxidative stress in diabetic rats by interfering with NADPH oxidase activity [201]. In addition, Chenguang et al. also found that empagliflozin suppressed oxidative stress and fibrosis via the inhibition of the transforming growth factor *β*/Smad pathway and activation of Nrf2/ARE signaling [202]. Furthermore, in diabetic mice, the GC-cGMP-PKG pathway was activated by empagliflozin, coming with alleviation of cardiac hypertrophy, reduced cardiac fibrosis, oxidative stress, and cell death [203]. Increased cGMP levels could help protect against cardiac fibrosis and hypertrophy and various agents such as cGMP-binding phosphodiesterase type 5 (PDE5) [204, 205].

Another FDA-approved agent related to oxidative stress is the soluble guanylate cyclase stimulator vericiguat [206]. Substantial evidence indicated that diastolic cardiac dysfunction is associated with excessive ROS production from the coronary microvasculature, decreasing cGMP levels, and PKG activity [207]. A recent clinical trial found that patients with diastolic cardiac dysfunction receiving vericiguat have an improvement in quality of life [208]. In the future, more studies should be carried out to identify ROS targets and develop successful therapies for the diseased heart. More details are necessary to uncover the mechanisms of SGLT2 inhibitors and vericiguat in MSC.

Animal experiments identified NOX4 as a potential therapeutic target [209]. NOX regulation could maintain the mitochondrial ROS homeostasis. Various NOX4 inhibitors such as GKT-831 are developed [210], although the effects of NOX inhibitors in reducing cardiovascular complications in diabetic patients need more tests. It is clear that mitochondria are the major ROS producers, Ting et al. employed mito-TEMPO, a mitochondria-targeted antioxidant, in the mice model of DCM and found the cardioprotective effects of mito-TEMPO [211]. Metformin is a widely used antidiabetic agent, which is also identified as a potent autophagy inducer. It has been proved in OVE26 mice that metformin could activate the AMPK pathway, enhancing mitochondrial autophagy and preventing cardiomyopathy [212]. Thus, the activation of AMPK may act as a novel approach for DCM treatment. Several molecules designed by targeting heme oxygenase-1 and mitochondrial aldehyde dehydrogenase (ALDH2) may serve as activators of AMPK, recovering normal autophagic activity and protecting from cardiomyopathy [162]. AICAR is an AMPK activator which was found to reduce blood glucose concentration and increase insulin sensitivity; however, AICAR was reported not suitable for clinical application [213, 214].

Many studies have investigated the efficacy of agents to prevent adverse cardiac remodeling in diabetic models. Cinnamoyl anthranilate could inhibit the TGF*β* pathway [215]. FT23 and FT011 are agents derived from cinnamoyl anthranilate, which could attenuate cardiac dysfunction in mice with DCM [216, 217]. cGMP homeostasis is a key factor to maintain PKG activity and thereby prevent cardiac stiffness [218]. Relaxin is a kind of antifibrotic hormone and exerts antifibrotic properties [219]. It has been reported that relaxin plays a critically protective role in various cardiovascular diseases, including suppression of inflammation [220]. Particularly, relaxin alleviated cardiac fibrosis by inhibiting collagen production from cardiac fibroblasts, fibroblast to myofibroblast transition, and increasing MMP levels [221]. Relaxin^−/−^ mice exerted relief of ventricular chamber stiffness and improved diastolic function after receiving recombinant human relaxin [222, 223]. A clinical trial of PDE5A carried out in patients with diabetes and diastolic cardiac dysfunction [224] found that sildenafil could improve LV torsion, strain, and contraction, reducing the levels of the pro-inflammatory factors C- X-C motif chemokine 10 and IL-8 in plasma [225].

GLP1 is an incretin secreted from intestinal L-type cells and reported to reduce blood glucose levels in a glucose-dependent manner by promoting insulin secretion. Zhou et al. reported that liraglutide, a GLP1 analog, is protected against the development of diabetic cardiomyopathy via inhibiting ER stress [226]. In addition, liraglutide could protect cardiomyocytes from IL-1*β*-induced mitochondrial membrane potential reduction and reduced ATP production [227]. In 2019, a clinical trial enrolled patients with diabetes treated with liraglutide demonstrated reduced early LV diastolic filling and LV filling pressure [228]. Conclusively, GLP1 receptor agonists are recommended as the first-line injectable agent for patients with diabetes and cardiovascular diseases [229].

In summary, various agents were developed as potential therapeutic strategies for MSC, and some have been proved to regulate mitochondrial function. However, there are still many unsolved questions needed to answer. More efforts are needed to facilitate the translation of basic research to the clinic.

## 5. Conclusion

Mitochondrial homeostasis is important for heart function. In this review, we summarized the evidence that mitochondrial disorder is a major cause of MSC. Many mechanisms including mitochondrial ROS, mitochondrial dynamics, and calcium disorder play great roles in MSC. Accordingly, many targets involved in mitochondrial homeostasis provide opportunities for the development of novel therapeutics. Although there are currently few drugs with successful clinical transformations, more researches are necessary to carry out and elucidate more promising targets in the future.

## Data Availability

All related materials are available from Hongwei Li.

## Abbreviations

AGEs: Advanced glycation end products
ALDH2: Aldehyde dehydrogenase
AMPK: Adenosine monophosphate activated protein kinase
ARE: Antioxidant response element
BMI: Body mass index
CAD: Coronary artery disease
CVD: Cardiovascular diseases
DCM: Diabetic cardiomyopathy
DM: Diabetes mellitus
ETC: Electron transfer chain
ER: Endoplasmic reticulum
FA: Fatty acid
GLP-1: Glucagon-like peptide-1
GSK-3*α*: Glycogen synthase kinase 3*α*
HF: Heart failure
HFD: High fat diet
H_2_O_2_: Hydrogen peroxide
IMM: Inner mitochondrial membrane
LCAD: Long chain acyl-CoA dehydrogenase
LVH: Left ventricular hypertrophy
MAOs: Monoamine oxidases
MAMs: Mitochondrial associated membranes
MCU: Mitochondrial calcium uniporter
MMPs: Matrix metalloproteinase
mPTP: Mitochondrial permeability transition pore
MSCDs: Metabolic syndrome–associated cardiac diseases
MSC: Metabolic syndrome–associated cardiomyopathy
NOX4: NADPH oxidase 4
Nrf2: Nuclear factor 2 related factor 2
PDC: Pyruvate dehydrogenase complex
PDK4: Pyruvate dehydrogenase kinase 4
PKC: Protein kinase C
PINK: PTEN-induced putative kinase 1
PPAR: Peroxisome proliferators activate receptors
ROS: Reactive oxygen species
SGLT2: Sodium-glucose cotransporter-2
SIRTs: Sirtuins
STZ: Streptozotocin
UCPs: Uncoupling proteins.
